# The road to breast cancer screening with diffusion MRI

**DOI:** 10.3389/fonc.2023.993540

**Published:** 2023-02-21

**Authors:** Mami Iima, Denis Le Bihan

**Affiliations:** ^1^ Department of Diagnostic Imaging and Nuclear Medicine, Kyoto University Graduate School of Medicine, Kyoto, Japan; ^2^ Department of Clinical Innovative Medicine, Institute for Advancement of Clinical and Translational Science, Kyoto University Hospital, Kyoto, Japan; ^3^ NeuroSpin, Joliot Institute, Department of Fundamental Research, Commissariat á l'Energie Atomique (CEA)-Saclay, Gif-sur-Yvette, France

**Keywords:** breast cancer, screening, breast imaging, diffusion MRI, standardization, screening techniques, cost, dedicated units

## Abstract

Breast cancer is the leading cause of cancer in women with a huge medical, social and economic impact. Mammography (MMG) has been the gold standard method until now because it is relatively inexpensive and widely available. However, MMG suffers from certain limitations, such as exposure to X-rays and difficulty of interpretation in dense breasts. Among other imaging methods, MRI has clearly the highest sensitivity and specificity, and breast MRI is the gold standard for the investigation and management of suspicious lesions revealed by MMG. Despite this performance, MRI, which does not rely on X-rays, is not used for screening except for a well-defined category of women at risk, because of its high cost and limited availability. In addition, the standard approach to breast MRI relies on Dynamic Contrast Enhanced (DCE) MRI with the injection of Gadolinium based contrast agents (GBCA), which have their own contraindications and can lead to deposit of gadolinium in tissues, including the brain, when examinations are repeated. On the other hand, diffusion MRI of breast, which provides information on tissue microstructure and tumor perfusion without the use of contrast agents, has been shown to offer higher specificity than DCE MRI with similar sensitivity, superior to MMG. Diffusion MRI thus appears to be a promising alternative approach to breast cancer screening, with the primary goal of eliminating with a very high probability the existence of a life-threatening lesion. To achieve this goal, it is first necessary to standardize the protocols for acquisition and analysis of diffusion MRI data, which have been found to vary largely in the literature. Second, the accessibility and cost-effectiveness of MRI examinations must be significantly improved, which may become possible with the development of dedicated low-field MRI units for breast cancer screening. In this article, we will first review the principles and current status of diffusion MRI, comparing its clinical performance with MMG and DCE MRI. We will then look at how breast diffusion MRI could be implemented and standardized to optimize accuracy of results. Finally, we will discuss how a dedicated, low-cost prototype of breast MRI system could be implemented and introduced to the healthcare market.

## Highlights

Screening has been shown as an effective method to improve the outcome of breast cancer, the leading cause of cancer in women. Mammography is the preferred method due to its low cost and favorable benefit/risk ratio. However, mammography has some limitations, such as exposure to X-rays, difficulty of interpretation in dense breasts, and overdiagnosis. Among other imaging methods, MRI has clearly the highest sensitivity and specificity. Still, MRI is mainly used to manage suspicious lesions revealed by mammography and not for screening, except for a category of well-defined women at risk, due to a high cost and a limited availability. While the standard breast MRI approach relies on the injection of contrast agents, which have their own contraindications, diffusion MRI which delivers information on tissue microstructure and tumor perfusion without the need for contrast agents, has been shown to provide a similar specificity and sensitivity, emerging as a promising alternative approach to breast cancer screening. To achieve this goal, it is necessary to standardize protocols for acquisition and analysis of diffusion MRI data. Second, the accessibility and cost-effectiveness of MRI examinations need to improve significantly, which may become possible with the development of dedicated breast, low-cost units for breast cancer screening.

## 1 Introduction

With the advent of widespread breast cancer screening by mammography (MMG) in the early to mid-1980s, detection of breast lesions has increased worldwide, and breast cancer is no longer a fatal disease when diagnosed and treated early. Approximately 60% of cancers diagnosed early have a 5-year survival of 99% after treatment and 31% have a 5-year survival of 85% (1). Breast cancer screening has therefore been shown to be an effective method of improving prognosis. In the absence of a reliable blood test, imaging is the primary approach available for screening. MMG has been the reference method until now because it is relatively inexpensive, widely available and has a favorable benefit/risk ratio with good sensitivity and specificity. Nevertheless, MMG suffers from certain limitations, such as exposure to X-rays given the recent discovery that breast tissue is more sensitive to the effects of radiation than most organs. In addition, with MMG, it is often not possible to predict on mammograms whether lesions are malignant, requiring active treatment, or not, so additional investigations must be performed, especially in dense breasts.

Of particular concern is the relatively high rate of overdiagnosis. Recent immunohistochemical studies have revealed that benign proliferative breast disease, most high-grade ductal carcinoma in situ (DCIS), and invasive carcinoma develop along distinct pathways, in contrast to colonic adenoma-carcinoma, which evolves along a single line (2). These findings suggest that different treatment approaches should be offered depending on the nature of the lesion, including therapeutic abstention for benign lesions. For example, while DCIS lesions often do not become invasive, patients diagnosed with DCIS are generally treated as if they were going to have invasive carcinoma. The rate of “overdiagnosis” is estimated to be between 21 and 66% (3). The social, ethical, and economic consequences of such management of DCIS lesions are enormous: more than 40% of women with DCIS undergo mastectomies, at a rate of some 10,000 per year, so much so that DCIS could be called a “mammographic disease” (4). Clearly, new approaches must be sought to better predict the grade and outcome of diagnosed breast lesions and to reduce burdensome, costly, and potentially unnecessary surgical procedures, such as mastectomy or axillary lymph node excision, whose morbidity is not negligible. It would also reduce surgical scars that could lead to pseudo-lesions on subsequent imaging. Conversely, the sensitivity of MMG for early detection of cancer in breast cancer screening is only 33% (40% for ultrasound) in patients with a high familial risk for breast cancer (lifetime risk ≧ 20%), missing some prognostically important diseases (5). Borderline lesions with uncertain malignant potential at biopsy [histologically classified as “B3”, (6)] most often result in a benign end result. However, these lesions are sometimes associated with the simultaneous presence of a malignant tumor with an enhancement rate of between 10 and 35%, and may also act as a risk factor or precursor to malignancy (7, 8). It is therefore necessary to obtain a more accurate classification of lesions at the time of initial diagnosis in order to personalize the therapeutic approach, avoid unnecessary procedures and reduce costs and social burden. With MMG, it is possible to suspect high-grade lesions from the morphology of microcalcifications, but grading is still difficult, with sparse biopsy sampling, because high-grade and low-grade components can coexist in the same patient or even in the same duct. Indeed, MMG may tend to detect slow-growing cancers.

Recently, breast MRI has been successfully introduced in the management of breast cancer. For example, in DCIS, the sensitivity of MRI for accurate assessment of the extent of DCIS is as high as 89%, much higher than MMG, tomosynthesis, or ultrasound (9). Increasing evidence suggests that, overall, breast MRI may be more sensitive, especially for the diagnosis of high-grade DCIS. Breast MRI is often performed by injection of gadolinium-based contrast agents (GBCA), but more recently, diffusion MRI, a completely noninvasive approach that is highly sensitive to changes in tissue microstructure, has been introduced for cancer imaging. Diffusion MRI has both very high sensitivity and specificity for the detection of breast malignancy (10). Diffusion MRI has been successfully used to differentiate between benign and malignant lesions of the breast, as well as tumor extension.

Yet MRI is exceptionally used for breast cancer screening, although supplemental MRI screening in women with extremely dense breast tissue and normal results on MMG has been recommended, as the addition of MRI leads to significantly fewer interval cancers than MMG alone during a 2-year screening period (11). Still, the main problem with breast MRI is that examinations are today performed using expensive general purpose MRI scanners. MRI is therefore performed as a second-line procedure, which adds to the cost of other imaging modalities (MMG and ultrasound), or in specific populations of women. In addition, there are concerns about the side effects of GBCA when performing dynamic contrast-enhanced (DCE) breast MRI (12). Blood tests may become available to screen for certain breast cancers, but they remain largely non-specific today with many false positives or negatives, and imaging will always remain mandatory to localize lesions and personalize treatment. If a dedicated, small-scale, inexpensive breast MRI scanner can be made available, it could be envisaged that one day MRI could be used as a screening imaging modality, instead of MMG, at least for a larger number of women at moderate to high risk based on personal history, genetic predisposition, or positivity to blood screening tests when these tests become reliable. This view was enthusiastically supported by an international (EU, USA, Asia) committee of breast imaging experts appointed by the European Society of Breast Imaging (EUSOBI) under the chairmanship of Profs. Denis Le Bihan and Julia Camps-Herrero (13). Breast cancer screening represents a huge market. In the United States alone, more than 60 million women over the age of 40 are responsible for 40 million mammograms per year, which corresponds to 65% of the population concerned (14). In contrast, MRI (using standard whole-body systems) accounted for only 0.4% of women aged 25-64 years in 2017 (15).

In this article, we will first review the principles and current status of diffusion MRI of the breast, and evaluate its clinical performance compared with MMG and DCE MRI. We will then discuss how diffusion MRI of the breast could be implemented and standardized to optimize accuracy of results. Finally, we will discuss how a dedicated, low-cost prototype breast MRI system could be implemented and introduced to the healthcare market.

## 2 Current place of breast MRI in the global management of breast cancer

**Figure 1:**
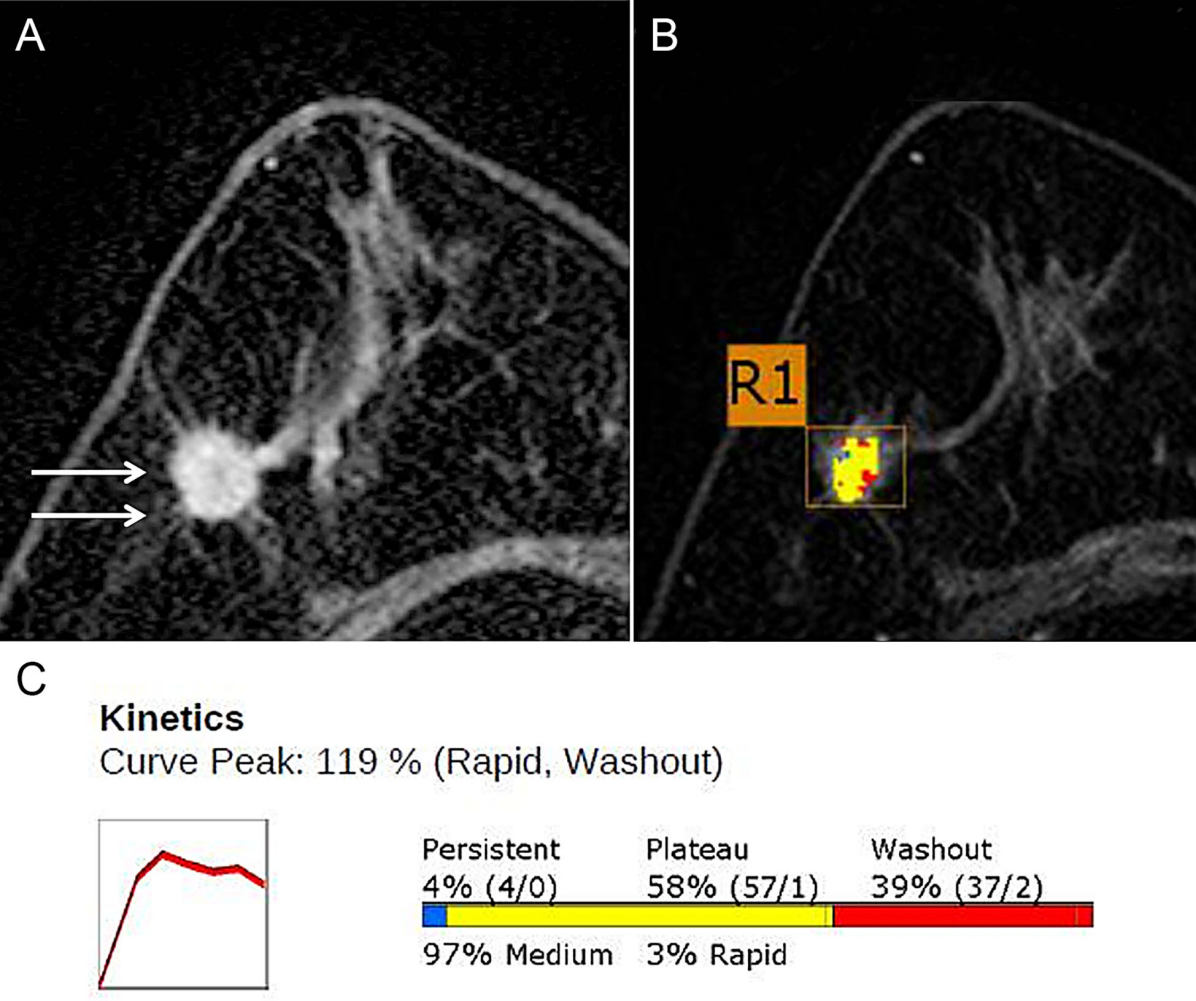
DCE-MRI in a 71-year-old Woman with grade 2 invasive ductal carcinoma in the right breast. **(A)** Axial contrast-enhanced T1w MRI image shows a 15-mm irregular mass (arrows). **(B)** Color axial maximum-intensity-projection MR image overlaid over the R1 breast mass. A computer-aided detection (CAD) algorithm displays areas in red, yellow, and blue indicating rapid washout-type delayed enhancement, plateau-type delayed enhancement, and persistent-type delayed enhancement patterns, respectively. **(C)** Graph of the contrast agent uptake shows a rapid initial enhancement and a rapid washout-type curve. The initial peak enhancement value was 119%. With respect to the delayed phase enhancement, 39% of the mass showed washout, 4% of the mass showed a persistent-type curve, and 58% showed a plateau-type curve. Adapted from (18).

Breast MRI has been widely available after the introduction of the use of contrast agents (16). Almost all types of breast cancer show detectable patterns of neovascularization with GBCA, which can readily extravasate into the extravascular and extracellular space (17). Thus, the likelihood of breast cancer can be considered extremely low in the absence of contrast enhancement. In practice, contrast-enhanced T1-weighted MRI is the gold standard. Many malignant breast lesions show maximal contrast enhancement in the early phase after injection, with GBCA being removed from the tissue in the late stage (**Figure 1**). Conversely, benign lesions and normal fibroglandular tissue usually show maximal enhancement in the late stage and of lower amplitude than in malignant lesions, allowing differentiation of these lesions (19). Given the high sensitivity for detection of breast cancer compared to other modalities such as MMG and ultrasound, breast MRI is also used for preoperative evaluation and tumor staging prior to treatment planning, monitoring tumor response to neoadjuvant therapies, to sort scars from recurrences, or in the presence of implants (20, 21).

However, despite its good clinical performance, MRI is usually performed in second intention. Breast cancer screening by MRI is therefore reserved for women with a moderate to high risk of breast cancer (personal history, genetic predisposition, follow-up after breast conserving surgery or contralateral breast screening, mediastinal irradiation, as for Hodgkin’s disease, suspicion of specific lesions, such as atypical ductal hyperplasia (ADH), atypical lobular hyperplasia (ALH), and lobular carcinoma *in situ* (LCIS) (22, 23). A first problem is the cost of MRI scans. Efforts have been made to shorten their duration [shortened DCE protocols, (24)]. However, there are also questions regarding GBCA-related side effects. The primary concern regarding nephrogenic systemic fibrosis (NSF) has almost disappeared, at least for DCE after assessment of renal function (25, 26), as it occurred only in patients with impaired renal dysfunction, and only seven of the 639 cases of patients with biopsy-confirmed NSF to date were discovered after 2008 (with the avoidance of the use of double and triple doses of GBCA that could trigger NSF) (27). The second concern is related to gadolinium retention in tissues, particularly the brain, after repeated exposure to GBCA (28). This risk is particularly important when considering the repeated annual injection of GBCA that would be required for screening (29). Various new approaches are being investigated to mitigate this risk, such as reducing the dose of GBCA. In a recent study, all breast cancers in 41 consecutive women with biopsy-proven breast cancer were detected as small as 0.4 cm with half (0.05 mmol/kg) a dose of gadobutrol on 3T DCE breast MRI (30).

Thus, there is a growing trend toward the use of new approaches based on unenhanced breast MRI for cancer detection (29). Although no consensus has yet been reached, these approaches could open up breast cancer screening to women at intermediate or even low risk for breast cancer. Given its high potential, diffusion MRI would be the obvious candidate for such an approach.

## 3 Breast diffusion MRI

### 3.1 Principles

#### 3.1.1 Diffusion-weighted imaging, DWI, and the apparent diffusion coefficient

While the concept of diffusion MRI emerged in the mid-1980s, diffusion MRI has become a mainstay of modern clinical imaging. Diffusion MRI is both a powerful method and concept because diffusing water molecules provide unique information about the microscopic architecture of tissues. Water diffusion is significantly diminished in most malignant tissues, and diffusion MRI, which requires no tracer injection, is rapidly becoming the modality of choice for detecting, characterizing, or even classifying malignant lesions, especially in the breast (31). Diffusion MRI is deeply rooted in the concept that, during their diffusion-induced movements, molecules probe the structure of tissues at a *microscopic* scale, well beyond the usual *millimeter* resolution of images. During typical diffusion imaging times of about 50-100 ms, water molecules move through tissues on average over distances of about 1-15 μm, bouncing off, passing through, or interacting with many tissue components, such as cell membranes, fibers, or macromolecules. Due to the tortuous movement of water molecules around these obstacles (“hindered” diffusion), the actual diffusion distance is reduced compared to free water. Therefore, non-invasive observation of water diffusion-induced displacement distributions *in vivo* provides unique clues to the fine structural features and geometric organization of cells in tissues, as well as to changes in these features as a function of physiological or pathological states.

**Figure 2:**
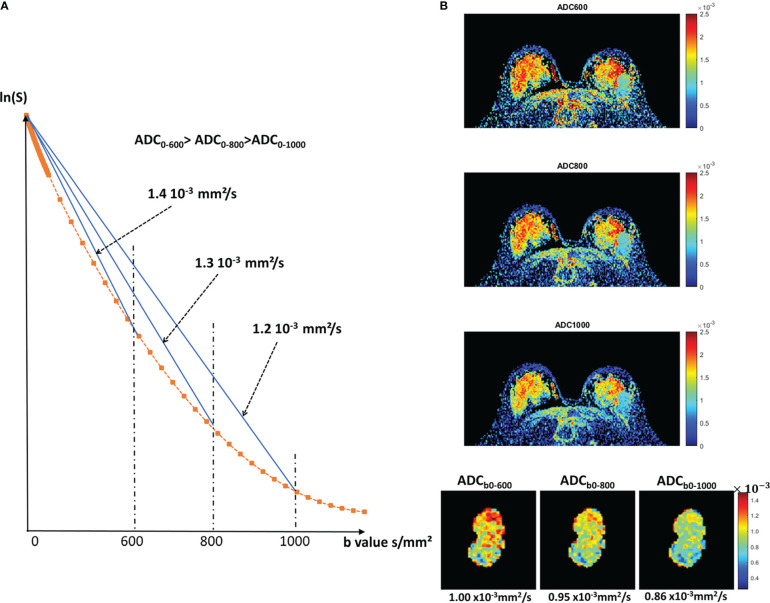
Diffusion attenuation versus b value. **(A)** Signal attenuation as a function of b value (logarithmic scale). With free diffusion we expect a straight line, whose slope is the diffusion coefficient. In tissues, diffusion is not free (non-Gaussian), resulting in a curvature. Therefore, the ADC taken from b=0 and any b values will decrease when b increases. The effects of IVIM, which result in a curvature at very low b values (<200s/mm²), are not shown for clarity. **(B)** Example of a breast tumor showing that indeed the ADC value decreases when using larger b values (reprinted with permission from 32).

MRI signals can be sensitized to diffusion by applying a pair of sharp magnetic field gradient pulses, the duration and separation of which can be adjusted to achieve a specific level of diffusion sensitization defined as the “b-value.” By acquiring data with different gradient pulse amplitudes, images with different degrees of diffusion sensitivity are obtained. The overall effect of diffusion in the presence of these gradient pulses is a signal attenuation and the MRI signal becomes “diffusion weighted”, hence the term “Diffusion Weighted Imaging” (DWI). The signal attenuation is more pronounced when large values of b are used and when diffusion is fast (because molecules diffuse over larger distances) (**Figure 2**). It is important to note that only the displacement (diffusion) component in the direction of the gradient pulses is detectable, but the diffusion can be anisotropic.

In DWI, *qualitative* contrast depends not only on diffusion, but also on other MRI parameters, such as T1 and T2 water relaxation times, which can lead to well-known artifacts, such as the “T2-shine-through” effect, as high T2 signal lesions (e.g., necrosis, cysts) can retain a relatively high signal level at high b values. Therefore, these images are often combined numerically to determine a *quantitative* estimate of the diffusion process in each image location, through an *Apparent Diffusion Coefficient* (ADC), “apparent” because diffusion is impeded by many processes (33):


(1) $$\text{ADC}=\text{ln}[\text{S}({\text{b}}_{0})-\text{S}({\text{b}}_{1})]/({\text{b}}_{1}-{\text{b}}_{0})$$

where S(b_0_) and S(b_1_) are the signals (in a voxel or region of interest, ROI) acquired at the b values b_0_ and b_1_, respectively. This simple ADC is an incredibly robust and powerful parameter, which has been widely used in all clinical applications of diffusion MRI since its inception (34). The optimal value of b_1_ that provides the best contrast-to-noise ratio in breast tissue, i.e., sufficient attenuation of the signal by scattering while maintaining a sufficient signal level is about 800s/mm² (13).

#### 3.1.2 Perfusion and IntraVoxel incoherent motion

Beyond molecular diffusion, blood microcirculation in capillary networks (perfusion) also contributes to the diffusion MRI signal. Indeed, the flow of blood water in pseudo-randomly oriented capillaries (at the voxel level) mimics a random walk (“pseudo-diffusion”) which leads to an attenuation of the signal in the presence of diffusion encoding gradient pulses. This effect has been named IntraVoxel Incoherent Motion (IVIM) (35). In the presence of blood microcirculation, the global attenuation of the MRI signal, S(b)/S(0), becomes the sum of two components, one for tissue diffusion and one for the blood compartment:


(2) $$\text{S}(\text{b})/{\text{S}}_{0}={\text{f}}_{\text{IVIM}}\text{ exp [}-\text{b}(\text{D}*+{\text{D}}_{\text{blood}})]+(1-{\text{f}}_{\text{IVIM}})\text{ exp (}-\text{bD)}$$

where f_IVIM_ is the fraction of circulating blood, D* is the pseudo-diffusion coefficient attributed to the random microcirculation of blood, D is the diffusion coefficient of water in tissue, and D_blood_ is the diffusion coefficient of water in blood. The perfusion effect is observed only at low values of b, because the pseudo-diffusion coefficient, D*, associated with blood flow is higher than the water diffusion coefficient and decreases more rapidly with the b-value.

IVIM MRI has become an important modality for perfusion imaging, with applications throughout the body (31, 36), particularly in cancer imaging (detection of neovascularization and treatment efficacy). A key feature of IVIM diffusion MRI is that it does not involve contrast agents, and it may serve as an attractive alternative to perfusion MRI in some patients with contraindications to contrast agents, or in patients with renal insufficiency at risk for NSF (see above).

#### 3.1.3 Non-Gaussian diffusion

Another important feature of diffusion MRI, which should be considered, counter-intuitively, as an advantage and not as a limitation, is that the ADC value depends on the acquisition parameters, especially the b-value, because diffusion in tissues is not “free” but “hindered”. With free (or “Gaussian”) diffusion, as in a cyst, the ADC remains the same regardless of the set of b values used to measure it (only the accuracy of ADC estimates changes with b values). However, in most tissues, the ADC value decreases as the diffusion sensitivity is increased by the b value (32) (**Figure 2**).

The reason is that an increasing number of molecules slowed down by their interaction with microstructural tissue components (fibers, cell membranes) during their diffusion movements become visible in the highly diffusion-sensitized MRI signal. This non-Gaussian diffusion behavior is therefore more pronounced when high b values are used. In short, sticking to the “optimal” b-value (e.g. 800s/mm²) deprives one of the potentially valuable clinical information about tissue microstructure encoded in the “non-Gaussian diffusion” provided by higher b-values. To reveal this hidden information about tissue microstructure, one must rely on models other than the standard ADC. There are essentially two types of such models. Some approaches aim to model the diffusion MRI signal biophysically, based on the different tissue compartments present in the tissue, as with NODDI (Neurite Orientation Dispersion and Density Imaging) used in the brain (37). The other way is simply to model the decay of the scattering signal mathematically, empirically, without any assumptions about the underlying biophysical properties of the tissue. Although several models have been proposed (38), the most popular approach simply quantifies the deviation of the scattering signal behavior from an ideal Gaussian behavior. This is the so-called Kurtosis model (39), also called Diffusion Kurtosis Imaging, DKI (40). With the Kurtosis model, which also includes the IVIM effect, the signal is described as follows:


(3) $$\text{S(b)}/{\text{S}}_{0}={\text{f}}_{\text{IVIM}}\text{ exp [}-\text{b}(\text{D}*+{\text{D}}_{\text{blood}})]+(1-{\text{f}}_{\text{IVIM}})\text{ exp }[-{\text{b ADCo+(b ADCo)}}^{2}\text{K}/6\text{] }$$

ADC_0_ is the extrapolated ADC value as b approaches 0 and K is the Kurtosis quantifying the deviation from Gaussian scattering (K=0 for Gaussian diffusion). Kurtosis has shown great potential for characterizing pathological or physiological conditions (41). A major drawback of DKI, however, is that it requires the acquisition of large data sets with multiple values of b to be fitted with equation (3), which significantly increases acquisition times, a premium in clinical practice.

#### 3.1.4 Abbreviated quantitative diffusion MRI protocols

**Figure 3:**
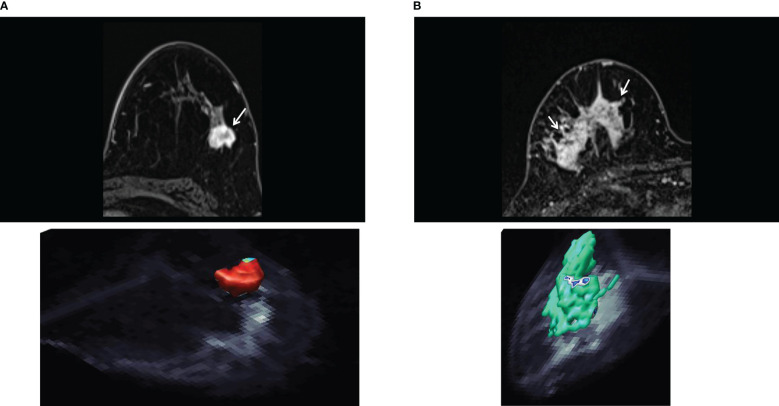
S-index. **(A)** Invasive ductal carcinoma of luminal A type in 50-year-old woman. The axial early-phase DCE-MRI image shows a mass with an irregular margin (top, arrow). The three-dimensional rendering voxel-by-voxel S-index image (bottom) shows the entire tumor in reddish color, corresponding to an average signature index (S-index) of this mass was 90.4. **(B)** Human epidermal growth factor receptor 2 enriched invasive ductal carcinoma in 73-year-old woman. The axial early-phase DCE-MRI image shows non-mass enhancement with a heterogeneous internal pattern in the right breast (top, arrow). The three-dimensional S-index rendering map of the entire tumor exhibits a yellow-green color (mean S-index of 55.8). [Adapted from (44)].

However, it is possible to obtain quantitative information about non-Gaussian diffusion with data sets acquired with a limited range of b values. For example, using data acquired for only 2 b values, one can calculate a shifted ADC (sADC). The concept of sADC (31) is based on the use of shifted key b-values (200 and 1500s/mm² for the breast, instead of 0 and 800s/mm²) providing an interesting balance between Gaussian and non-Gaussian diffusion effects. This approach has been evaluated for the breast (42). Another approach, S-index, provides a direct classification of tissue types by calculating a distance between the acquired signals and a library of reference (“signature”) signals from known or simulated tissues (e.g., benign, malignant, etc.) by intrinsically accounting for Gaussian and non-Gaussian diffusion effects, without the need for any mathematical or biophysical modeling (43). This approach has also been shown to provide the immunohistochemical status and molecular subtypes of invasive breast carcinomas (44) (**Figure 3**).

It is also possible to estimate the main parameters of the IVIM/Kurtosis model described by equation [3], f_IVIM_, ADCo and K, without fitting, using a limited set of 4 b values (b0, b1, b2 and b3 in ascending order), providing the signal:noise ratio is not too low. The proposed algorithm assumes that IVIM effects become negligible in signals acquired above b1 and that non-Gaussian diffusion effects appear visible in b2 and b3 signals. According to this 4b algorithm the model parameters estimates can be calculated as:


(4) $${\text{f}}_{\text{IVIM}}\approx 1-\text{exp[}-(\text{D}1-\text{D}2-\text{H})(\text{b}1.\text{b}2)/(\text{b}2-\text{b}1)]$$

(5) sADC=ln[S(b1)/S(b3)]/(b3−b1)

(6) ADCo≈sADC+(b1.b3) A

(7) $$\text{K}\approx 6\text{A}/{\text{ADCo}}^{2}$$

where D1=ln[S(0)/S(b1)]/(b1-b0); D2=ln[S(0)/S(b2)]/(b2-b0); D3=ln(S(0)/S(b3)/(b3-b0); H=(D2-D3)/(b3-b2)+ln(1-F)/(b3.b2); F= 1-exp[-(D1-D2)(b1.b2)/(b2-b1)]; A= (D2-D3)/(b3-b2)+ln(1- f_IVIM_)/(b3.b2).

In the absence of IVIM and non-Gaussian diffusion effects one obviously has D1=D2=D3=sADC, ADCo=sADC and K=0.

If non-Gaussian diffusion is present without IVIM effects (f_IVIM_=0) ADCo and K are obtained exactly as:


ADCo(no IVIM)=sADC+(D2−D3) (b1.b3)/(b3−b2)

$$\text{K(no IVIM)}=6(\text{D}2-\text{D}3)/[(\text{b}3-\text{b}2){\text{ ADCo}}^{2}]$$

**Figure 4:**
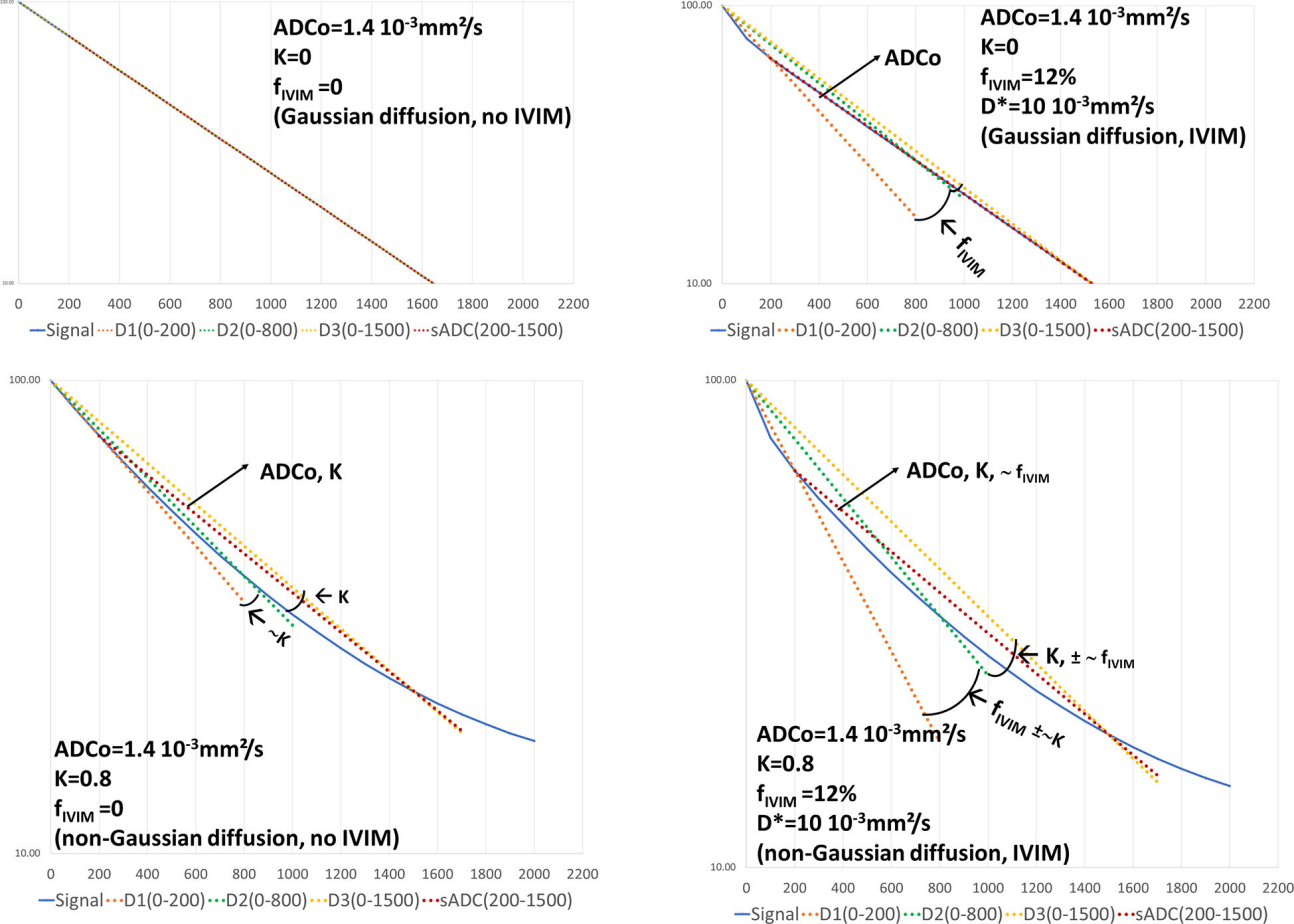
Graphical representation of the 4b-diffusion MRI abbreviated protocol. The plots show the signal attenuation and the straight lines associated to the intermediate calculation parameters (slopes) D1, D2, D3 used to estimate fIVIM, ADCo and K, as well as sADC. In the presence of free diffusion (K=0) and in the absence of IVIM effects (top left) the signal attenuation follows a straight line whose slope is ADCo. D1, D2, D3 and sADC are all equal to ADCo. When IVIM effects appear (top right) the D1 line starts to deviate from the signal attenuation curve with an angle with D2 reflecting f_IVIM_. With non-Gaussian diffusion effects only, both D1 and D3 deviate from D2 with an angle reflecting K (bottom left). The sADC line depends on ADCo and K. When both IVIM and non-Gaussian diffusion effects are present one can see that the angle between D1 and D2 primarily reflects f_IVIM_ while the D2D3 angle mainly reflects K (bottom right). The sADC now reflects ADCo, K and f_IVIM_. By combining sADC with D1, D2 and D3 one can get accurate estimates of ADCo, K and f_IVIM_ using equation (4-7).

A graphical interpretation of this set of equations can be given by plotting the (log) of the (curved) signal attenuation versus the b value and the straight lines corresponding to D1, D2, D3 and sADC (**Figure 4**). In the absence of IVIM and non-Gaussian diffusion effects the signal attenuation follows a straight line with a slope D1=D2=D3=sADC=ADCo. In the presence of IVIM effects only the curvature at low b values creates an angle between the D1 and D2 lines. From this angle f_IVIM_ can be estimated (Eq. 4) while ADCo remains very close to the sADC (Eq. 6). In the presence of non-Gaussian diffusion only the curvature at high b values forms an angle between the D2 and D3 lines, from which K can be estimated (Eq. 7). However, one can see that f_IVIM_ and non-Gaussian diffusion slightly contribute also to the angle between the D2 and D3 lines, and the angle between the D1 and D2 lines, respectively. Hence, estimated f_IVIM_ and K values must be corrected (variables H and A in Eq. 4, 6). Note that the sADC now includes ADCo, f_IVIM_ and K effects, so that the ADCo values derived from sADC must be corrected (A variable). Also, with this algorithm D* cannot be estimated, however, a review of the literature shows that D* is a parameter difficult to estimate even with the full fitting approach, resulting in extremely variable clinical relevance.

It is expected that those abbreviated quantitative DWI protocols will play a major role, in addition to qualitative DWI, such as DWIBS (see below), in the context of breast cancer screening with diffusion MRI.

#### 3.1.5 Diffusion tensor imaging

Molecular mobility in biological tissues may not be the same in all directions, which is referred to as diffusion anisotropy. In the breast diffusion anisotropy can arise from the geometric organization of the glandular tissue around ducts. To characterize the effects of anisotropy, diffusion-weighted images must be sensitized to diffusion along multiple directions (at least 6) within the Diffusion Tensor Imaging (DTI) (45) With DTI one gets information on the tissue mean diffusivity, MD, which is equivalent to an orientation invariant ADC, and lambda values (λ_1_, λ_2_, and λ_3_) which give diffusivity along the main diffusivity directions (so-called eigenvectors ϵ_1_, ϵ_2_, and ϵ_3_). The eigenvector ϵ_1_, associated with the highest λ value, λ_1_, is aligned along the main orientation of aligned structures (e.g. ducts), allowing to produce maps showing their orientation in space. Some vendors propose to estimate the MD from a set of 3 orthogonal directions, but this is an approximation that should not be used in the presence of strong anisotropy effects. The genuine mean diffusivity is simply the average of the 3 λ values. The other important parameter, called Fractional Anisotropy (FA), quantifies the degree of anisotropy (FA = 0 indicates that diffusion is isotropic). It is calculated from the λ values. Whereas the existence of diffusion anisotropy in fibroglandular breast tissue has been claimed by many groups (46–49), the nature of the anatomical features which might cause this anisotropy remains somewhat controversial. Some studies have shown that breast cancer lesions could be associated with significantly lower FA values relative to normal breast tissue, and that λ_1_ or (λ_1_–λ_3_) could overperform the ADC (or MD) for lesion detection and classification (50, 51). However, one has to keep in mind that λ_1_ and λ_3_ (and FA which depends on them) are, by principle, highly sensitive to noise because of the strongly non-linear nature of the DTI calculation algorithm. The mere fact that MD values are lower in malignant lesions than in normal tissue might lead to reduced FA values, which should not necessarily be interpreted as “reduced anisotropy” (32).

### 3.2 Clinical performance of breast diffusion MRI

There is an extensive literature on breast diffusion MRI. We give below a brief summary of the highlights. Many more details can be found in (10). In addition, a survey of the implementation of breast DWI in clinical practice from the EUSOBI has recently been published (52).

#### 3.2.1 Qualitative lesion detection

Most often diffusion MRI is used qualitatively for lesion detection. Lesion detection can be achieved from DWI acquired with high b values, which have a higher contrast between breast lesions (which appear bright) and normal parenchyma (dark background). Breast cancer detection using DWI has been shown to be more sensitive than MMG, with the DWI screening approach allowing to detect mammographically occult cancers (53–55) and DWI has been shown to detect significantly more contralateral breast cancers in women with unilateral breast cancer than MMG (56). High b-values are also useful in decreasing false-positive breast cancer cases (57).

A variant of the DWI techniques for qualitative lesion detection is DWIBS (Diffusion-weighted Whole-body Imaging with Background body signal). A previous study in 280 patients has shown that the diagnostic performance using non-contrast technique including DWIBS for breast lesion detection (sensitivity, specificity, diagnostic accuracy, Positive Predictive Value (PPV) and Negative Predictive Value (NPV) values of 94%, 79%, 86%, 79% and 94%, respectively) was comparable to that of DCE-MRI (sensitivity, specificity, diagnostic accuracy, PPV and NPV values of 98%, 83%, 90%, 84% and 98%, respectively) (58). DWIBS performed with Maximum Intensity Projection (MIP) mapping also has a comparable diagnostic performance (sensitivity, specificity, PPV and NPV values of 92%, 94%, 93%, and 92%, respectively) to that of DCE-MRI performed with MIP (sensitivity, specificity, PPV and NPV values of 85%, 90%, 89%, and 87%, respectively). MIP-DWIBS has been shown to rule out previously suggested malignancy on screening MMGs in 50 participants with carcinoma in 24 patients (59).

#### 3.2.2 Quantitative lesion evaluation

Nevertheless, a unique feature of breast DWI is its quantitative assessment capability. As the most popular quantitative marker, the ADC can be used as a threshold to sort out benign from malignant lesions (13, 60, 61), but also to build a lexicon to describe and classify lesions, for instance to distinguish breast cancers from benign lesions (13). Many groups have also found significant differences of ADC values between benign and metastatic breast lymph nodes (62–64), however, their diagnostic performance in differentiating these lymph nodes still need further investigation (64) compared to simpler markers such as the lymph node size. Quantitative DWI in addition to DCE‐MRI and other plain MRI such as T1WI and T2WI also leads to improved diagnostic performance, in terms of specificity for BI-RADS (Breast Imaging-Reporting And Data System) 3 and 4 lesions, or evaluating malignancies with BI-RADS 4 lesions (65, 66).

**Figure 5:**
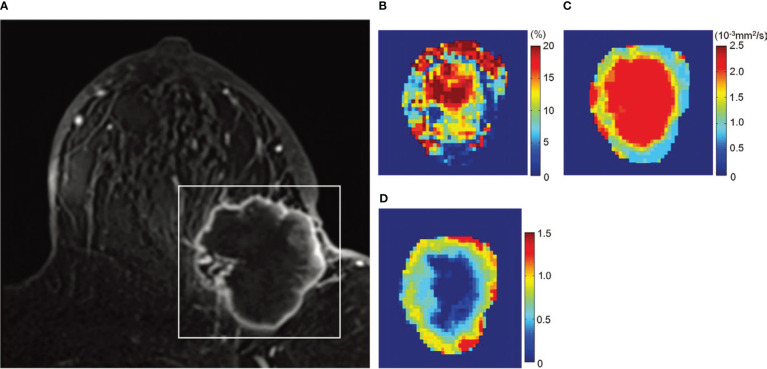
Example of non-Gaussian diffusion MRI maps in breast lesions. Images in a 72-year-old woman with invasive ductal carcinoma. **(A)** Dynamic contrast-enhanced axial MR image, **(B)** f_IVIM_ map, **(C)** ADC_0_ map, and **(D)** K map. The white rectangle on **(A)** shows the area covered by the parametric maps. **(B)** f_IVIM_ distribution is heterogeneous inside the tumor. The peripheral area of the tumor exhibits low ADC_0_ **(C)** and high K values **(D)**, suggesting high cellularity diffusion hindrance effect (likely from cellular membranes) corresponding to the viable malignant component (also high f_IVIM_ values), whereas the central part shows high ADC_0_ and low K, suggesting lower cellularity (possible necrosis with fluid motion at the center resulting in high f_IVIM_ values). [Adapted from (42)].

DWI is often used in multiparametric protocols in combination with other MRI modalities, such as DCE-MRI, contributing to improve overall diagnostic specificity and accuracy over DCE-MRI alone (67), especially when examining non-Gaussian diffusion (42) (**Figure 5**). The combination of DCE-MRI and DWI could increase diagnostic accuracy in characterization of non-mass-like enhancement lesions (68). It has also been reported that DWI combined with T2WI improved the diagnostic specificity of enhancing lesions incidentally detected in breast DCE-MRI (69), and that multiparametric DWI outcome parameters have associations with molecular prognostic factors or subtypes (70, 71).

Advanced diffusion markers (IVIM, Kurtosis, DTI) can further increase diagnostic performance, although there are not yet used in routine clinical practice (**Figures 3**, **5**). IVIM parameters have been shown to provide a high diagnostic performance in differentiating benign and malignant breast tumors (sensitivity = 86%, specificity = 86%, AUC = 0.91 for D, sensitivity = 80%, specificity = 76%, AUC = 0.85 for f, and sensitivity = 84%, specificity = 59%, AUC = 0.71 for D*) (72), especially in combination with DCE-MRI (73), and IVIM parameters are known to be correlated with DCE-MRI parameters (74). IVIM histogram parameters have been shown to be associated with molecular prognostic factors (75, 76). Regarding DKI higher K and lower MD values are usually observed in malignant compared to benign lesions (41, 77), DKI was found to be useful in the differentiation of additional suspicious lesions at preoperative breast MRI (78). In 2 recent meta-analyses of DKI studies (79, 80) the sensitivity and specificity of K and MD to differentiate malignant from brewing breast lesions were found to be around 89-90% and 86-88% for K, and 84-86% and 83-88% for MD. The utility of DKI in differentiating molecular prognostic factors (81) or predicting treatment response (82) has also been reported.

As for DTI parameters, malignant breast lesions have significantly lower MD and λ_1_ values compared to benign lesions (46). Indeed, λ_1_ and MD are known to have a high diagnostic performance in differentiating malignant and benign breast lesions (AUC 0.97, sensitivity 93%, specificity 92% for λ_1_ and AUC 0.92, sensitivity 87%, specificity 83% for D (50), although this trend might not be related to diffusion anisotropy [see above (32)]. Similarly, the use of FA to differentiate between malignant and benign breast tumors remains controversial, suggesting that caution should be exercised in the use of this parameter, although several studies have suggested its usefulness to sort out malignant and benign lesions (14, 50). Yamaguchi et al. (83) also reported higher FA in lesions with more favorable prognostic factors, such as positive estrogen receptor status, lower nuclear grade and cancer intrinsic subtype, and reduced DTI metrics had association with poor prognostic factors of breast cancer (84). A correlation between DTI parameters and molecular prognostic factors (estrogen receptor status or Ki-67) has been shown (49) and DTI has been investigated to differentiate recurrent breast cancer from post operative changes with breast-conserving surgery in patients (85).

#### 3.2.3 Diffusion MRI as a stand-alone modality

In reviewing the literature, the overall sensitivity of DWI alone is very high, approaching 90% for detection of breast malignancy (with a specificity of approximately 82%, superior to any other imaging modality, including GBCA MRI) (51).

Many investigators have studied the potential of DWI alone for non-contrast cancer detection. The performance of DWI imaging for cancer detection is variable across studies, with a mean sensitivity of 81% (range 44-97%) and a mean specificity of 88% (range 73-96%) (86). This variation could be due to the diversity of the study population as well as the image acquisition protocols, highlighting the need for standardization (see below). Nonetheless, DWI based primarily on qualitative assessment is less sensitive than DCE MRI (mean sensitivity of 80 vs. 90s % for DWI vs. DCE MRI in studies (54, 58, 59, 67, 87–93). This situation is entertaining the idea that diffusion MRI would be difficult as a stand-alone modality compared to DCE-MRI and multiparametric MRI (67). However, in reviewing the literature, the overall sensitivity of quantitative ADC alone is very high, approaching 90% for detection of breast malignancy (with a specificity of approximately 82%, superior to any other imaging modality, including GBCA-MRI) (51). In any case, with respect to screening, diffusion MRI offers much better clinical performance than MMG or ultrasound (94). This is an important point, considering that repeated use of GBCA would be a problem for screening. Not only does DWI remain more sensitive than MMG across studies (52, 92, 95, 96), but mammography-occluded breast cancers are better represented with DWI than with ultrasound (94).

## 4 How breast diffusion MRI could be implemented to give optimal performance in breast cancer screening

### 4.1 Standardization

Despite this good clinical performance, it may seem surprising that DWI has not yet been recommended to be used as a stand-alone modality for breast cancer evaluation, let alone for breast cancer screening. Diffusion MRI is not even included in the BI-RADS lexicon used to assess breast lesions from GBCA MRI (97), although it is considered useful (52). The main reason is likely the high variability of the results found in the literature, especially with regard to ADC values (13, 98). The EUSOBI international committee on breast DWI has provided guidelines for obtaining optimized and consistent results (13). This report, along with the EUSOBI survey (52) have pointed out to an urgent need for standardization of DWI acquisition and processing protocols to achieve consistent results among breast DWI users.

Technical advances in MRI scanners, particularly for gradient hardware and fast imaging, facilitate the exploration of new features beyond ADC by allowing perfusion-driven IVIM to become more reliable (99), providing access to non-Gaussian diffusion through high b-values, and investigating diffusion time effects. This increasing flexibility of diffusion MRI acquisitions is supporting the expansion of more complex models, allowing for a better understanding of the relationship between diffusion MRI parameters and the microscopic characteristics of the underlying tissue. This is particularly true in the field of breast imaging, where a wide variety of diffusion MRI techniques have great potential for clinical applications in the breast field. However, this flexibility implies that some normalization must be implemented in order to compare quantitative results obtained at multiple sites. Not only are ADC values strongly dependent on b-values (100–102), but they are also influenced by TE, due to differences in T2 values between tissue components. Hidden parameters, such as diffusion time (set by the duration and intervals of the gradient pulses) also have important effects. For example, while high performance gradient hardware can achieve high b-values with shorter TEs, increasing the signal-to-noise ratio, diffusion contrast may be partially lost, as diffusion hindrance decreases with short diffusion time (103). Thus, there is a clear need for standardization of acquisition protocols. Validation of these protocols in different clinical sites would benefit from calibrated phantoms, as suggested by EUSOBI (the European Society of Breast Imaging) (13), QIBA (Quantitative Imaging Biomarkers Alliance) organized by the Radiological Society of North America (RSNA) (104). Clearly, additional efforts are needed in collaboration with vendors if consensus is to be reached on optimal acquisition parameters for diffusion MRI of the breast (10).

### 4.2 Technical requirements and improvements

#### 4.2.1 Image acquisition

Single-shot echo-planar imaging (EPI) is currently the method of choice for *in vivo* diffusion imaging, as it allows efficient and ultrafast acquisition of multiple diffusion-weighted images (different b-values) without in-plane motion artifacts, to which diffusion MRI is notoriously sensitive. Nevertheless, EPI has several limitations related to spatial resolution, artifacts, and signal-to-noise ratio. In particular, small breast lesions (<2 mm) may be undetectable. In addition, EPI requires a very homogeneous magnetic field. For breast imaging, field inhomogeneities may be more pronounced at the air/tissue interface in the anterior part of the breast, resulting in local image distortion or signal loss. Another source of geometric distortion comes from eddy currents induced by the switching of strong diffusion encoding gradient pulses. Therefore, the degree of geometric distortion increases with the b-value. This geometric distortion must be corrected before performing any quantitative analysis involving multiple values of b to avoid artifacts around small lesions, especially at high spatial resolution. Segmented EPI acquisitions (e.g., ‘RESOLVE’ (Readout Segmentation of Long Variable Echo-trains) (105) can overcome these limitations at the cost of longer diffusion times and a sensitivity to motion between acquired segments that must be corrected using *ad-hoc* approaches during image reconstruction. Parallel acquisition techniques, which allow simultaneous signal collection using an array of multiple RF coils, can also address these limitations. Incorrect fat suppression can also lead to misinterpretation of diffusion MRI, as residual fat present in breast tumors results in low diffusion values, mimicking malignancy, visually and quantitatively (ADC values). The Spectrally Adiabatic Inversion Recovery (SPAIR) method has been recommended for breast imaging (13).

#### 4.2.2 Image processing

Efforts are also needed on the image processing side. Diffusion-weighted images are often noisy, especially for high b-values, because the signal is strongly attenuated by the diffusion effect. Noise is a vicious enemy because it is not always visible, while having a profound impact on the values of the parameters estimated with the various models available including ADC. For high b-values, due to the nature of the MRI signal (a “magnitude” signal that cannot be negative), there always remains a background noise signal and the diffusion signal remains above a threshold, the “noise floor”, instead of asymptotically approaching 0, resulting in underestimated ADC values. If one classifies lesions (e.g., benign or malignant) on the basis of ADC threshold values, it is easy to see that this trap of underestimated ADC could lead to a significant bias toward the “malignant” nature of lesions. Therefore, an adequate signal-to-noise ratio must be ensured, e.g., by increasing the voxel size (at the expense of spatial resolution) or by repeating image acquisitions at high b-values for signal averaging before amplitude reconstruction (which unfortunately increases acquisition time). Finally, background noise effects must also be removed from the signals before analysis, especially in images acquired at high b-values (106). Noise effects may partly explain the discrepancies in the literature on the different reported values of diffusion MRI and IVIM parameters. Image preprocessing could also include steps to correct for motion artifacts and geometric distortion before the signals can be processed to calculate ADC values or estimate parameters for advanced DWI models. Another problem with clinical diffusion MRI is that quantitative analysis is often performed remotely on workstations and not on the acquisition console, which is cumbersome. Efforts are underway by vendors to provide dedicated tools for breast DWI [see the final chapters of the book (10)].

DWI data analysis would also benefit from recent developments in artificial intelligence (AI). Various approaches are being investigated for breast MRI, as well as remarkably increasing applications of convolutional neural network models (107) and machine learning (108). For example, a recent study showed that DWI radiomic classifiers for differentiating suspicious lesions in 50 asymptomatic women screened with MMG outperformed the average ADC, with an area under the curve (AUC) of receiver operating characteristics (ROC) of 84.2%/85.1% for unconstrained/constrained radiomic classifiers compared with 77.4% for the average ADC (109). The AI-based multiparametric MRI approach, including DCE, T2WI, and DWI, had better diagnostic performance (AUC ROC area of 0.852) than ultrafast DCE alone (0.811) (110). Machine learning with multiparametric MRI (DCE, DWI, and T2WI) also found that several features, including those of DWI (minimum ADC), were relevant features for predicting residual cancer burden (111). Whole breast segmentation on DWI data from different institutions and scanner types was also found to be effective using deep learning methods, which could facilitate computer-assisted quantitative analyses of DWI images of the breast (112).

### 4.3 Toward a low-cost, dedicated MRI system for breast cancer screening

Given the outstanding clinical performance of breast MRI, which has much higher sensitivity and specificity than MMG and does not rely on x-rays, it should ideally be the screening modality of choice for many women. Unfortunately, breast MRI remains expensive when performed using general-purpose body MRI scanners operating at 1.5T or even 3T. The cost (and limited availability) of these scanners prohibits the use of MRI as a screening modality (the cost today is approximately $1000 for a 40-minute exam). Breast cancer screening with MRI is therefore reserved for women with moderate to high risk of breast cancer, as detailed above. However, if a small-scale, inexpensive, dedicated breast MRI scanner were available, MRI could be used as a screening imaging modality, rather than MMG, for more women, such as women with dense breasts or a family history of breast cancer.

One issue that comes to mind when considering breast cancer screening with MRI is the use of GBCA, as examinations will need to be repeated over many years, knowing that an accumulation of gadolinium deposits in the brain or other organs in patients who have received multiple injections of contrast agents has been demonstrated. For this reason, several groups have considered the possibility of using diffusion MRI as a stand-alone imaging modality for breast cancer screening (55, 59). As detailed above, diffusion MRI, which is completely noninvasive, has been successfully used to differentiate between benign and malignant breast lesions and tumor extension. Diffusion MRI also has the potential to detect many occult mammographic and clinical carcinomas of the breast, making it a preferred modality for cancer screening. Contrast agents could then still be used, but as a second line if necessary.

**Figure 6:**
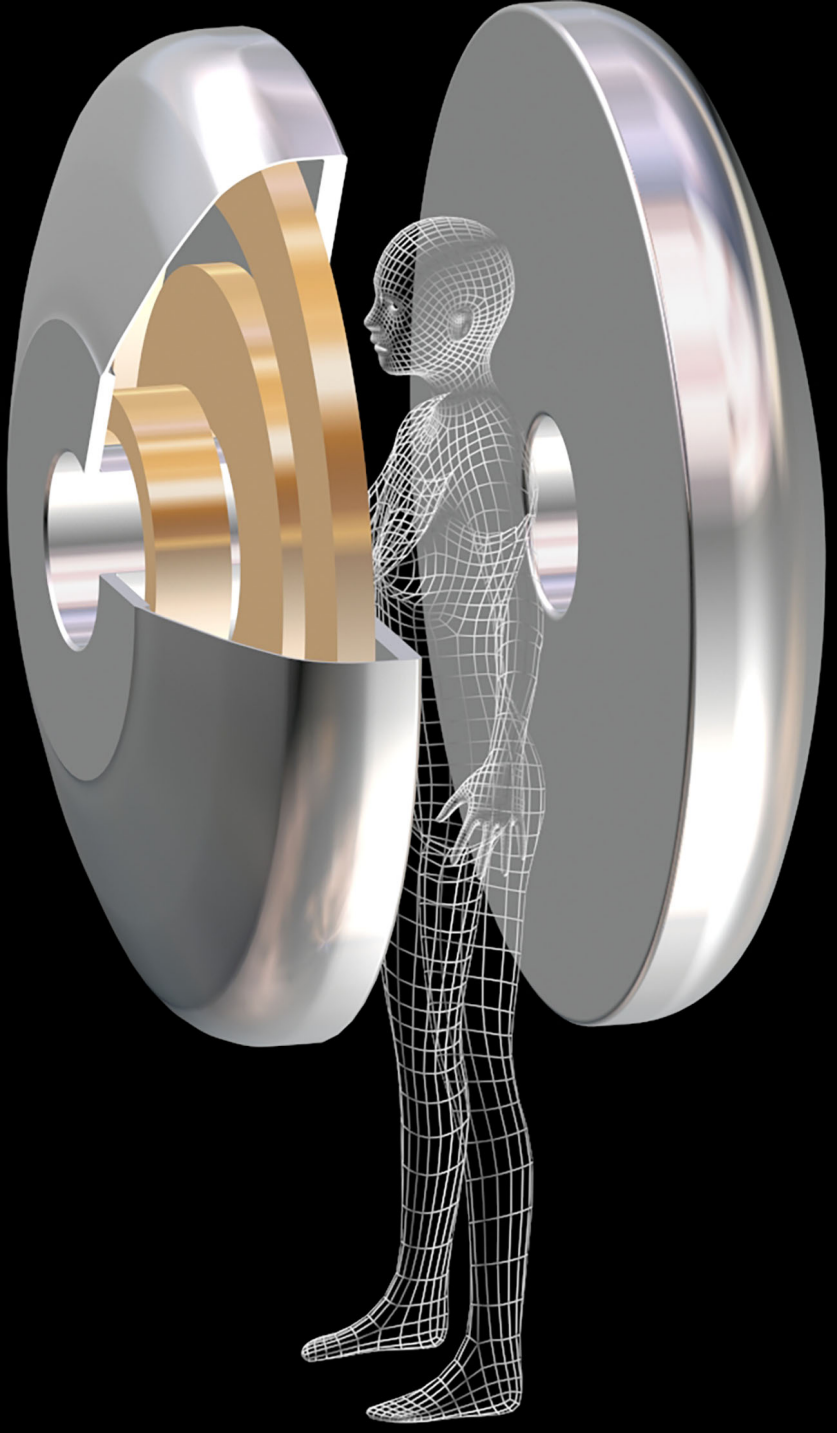
Prototype of a MRI magnet dedicated to breast cancer screening. The superconducting magnet consists in 2 halves. The patient stands between the 2 halves. To reduce size and cost, this magnet can be tailored for scanning one breast at a time (courtesy T. Schild, Irfu/CEA).

A major technical implication of using diffusion MRI instead of GBCA MRI is that only one breast can be scanned at a time, as with MMG, making the design of a dedicated breast MRI scanner much easier, smaller, and therefore available at much lower cost. Here we propose some specifications that might be kept in mind when designing such a dedicated imaging system. Ideally, the device should be small to be mobile and affordable. In total, the footprint of the system should also be small compared to the 5-gauss line. Patients could be in a standing position, as a bed structure would increase space and cost (**Figure 6**). This will also shorten the examination time and therefore reduce imaging costs. The disadvantage is that breast motion (which is already a problem with conventional MRI) will have to be controlled mechanically (motion sensors) and/or using *ad hoc* post-processing algorithms. Field homogeneity should be < 1ppm/20cm peak-to-peak (0.05 ppm after shimming). This is a very important requirement because breast MRI requires “fat suppression” techniques that rely on the differential frequencies between fast and water resonance frequencies. In addition, thoracic bones and air contained in the lungs are responsible for local magnetic susceptibility effects that distort the magnetic field. As with general MRI, the field stability must be better than 0.05ppm/h (10^-4^ppm/10 minutes). An open design will also allow image-guided biopsy or therapy (113). The field strength should be low to keep construction and maintenance costs as low as possible, ideally using helium-free magnets. This means that several technical improvements must be implemented to maintain sufficient signal-to-noise ratios, especially when using high diffusion weighting (large b values). Efficient and powerful gradient hardware must be implemented to achieve high b-values while maintaining a short TE. Innovative radio frequency systems will need to be designed for both transmission and reception. For example, receive coil arrays could be tailored to different breast sizes to maximize fill factor, such as “bra coils.” AI algorithms that have been developed for acquisition (sparse sampling) and signal processing (114) will help maintain adequate signal-to-noise levels while achieving spatial resolution greater than 2 mm. To exploit the full content of the diffusion MRI signal, one can even envision that processing will be performed not on the reconstructed images (which are only for the eyes of radiologists and clinicians), but on the denoised raw signals using AI algorithms trained and optimized to detect disease signatures. The images will then be reconstructed by focusing on these anomalies when they are detected. Assuming that no suspicious lesions will be found in the vast majority of cases, radiologists will be able to focus on the remaining cases that the AI system will identify as difficult to classify.

Clearly, designing such a prototype is a team effort. Clinicians must work closely with physicists, engineers and technicians, not only to design the most patient-friendly system, but also with market attention. The price of the overall system should be similar to that of high-end mammography systems, around 400 k€. In addition to the cost of building a proof-of-concept prototype, costs for patenting, multi-center trials and market research, calibration and quality control, FDA (US Food and Drug Administration) and CE (Conformité Européenne) marking, etc., must be considered. We sincerely hope that some vendors will be interested in this challenge, invest and bring such a breast MRI screening device to the market for the benefit of patients worldwide.

## 5 Conclusion

Non-contrast breast diffusion MRI has emerged as a potential alternative for breast cancer screening and lesion characterization. Without GBCA injections and with higher sensitivity and specificity than MMG, breast diffusion MRI is emerging as an ideal imaging modality for cancer screening. Consensus is needed to define the population categories that could benefit from this approach, such as women at moderate to high risk for cancer. Efforts are still needed to standardize acquisition and processing protocols and to decrease the cost of breast MRI examinations. To this end, the development of a low-cost MRI system dedicated to DWI for breast cancer screening is an option that should be seriously considered.

## Author contributions

Conception or design of the work. MI, DB. Materials collection. MI, DB. Materials analysis and interpretation. MI, DB. Drafting the article. MI, DB. Critical revision of the article. MI, DB. Final approval of the version to be published. MI, DB. All authors contributed to the article and approved the submitted version.
